# Workgroup Report: Implementing a National Occupational Reproductive Research Agenda—Decade One and Beyond

**DOI:** 10.1289/ehp.8458

**Published:** 2005-10-26

**Authors:** Christina C. Lawson, Barbara Grajewski, George P. Daston, Linda M. Frazier, Dennis Lynch, Melissa McDiarmid, Eisuke Murono, Sally D. Perreault, Wendie A. Robbins, Megan A.K. Ryan, Michael Shelby, Elizabeth A. Whelan

**Affiliations:** 1 National Institute for Occupational Safety and Health, Cincinnati, Ohio, USA; 2 Procter & Gamble, Cincinnati, Ohio, USA; 3 Department of Preventive Medicine and Public Health and Department of Obstetrics Gynecology, University of Kansas, Wichita, Kansas, USA; 4 Occupational Health Program, University of Maryland School of Medicine, Baltimore, Maryland, USA; 5 National Institute for Occupational Safety and Health, Morgantown, West Virginia, USA; 6 National Health and Environmental Effects Research Laboratory, Office of Research and Development, U.S. Environmental Protection Agency, Research Triangle Park, North Carolina, USA; 7 Center for Occupational and Environmental Health, University of California at Los Angeles, Los Angeles, California, USA; 8 Department of Defense Center for Deployment Health Research, San Diego, California, USA; 9 National Institute of Environmental Health Sciences, National Institutes of Health, Department of Health and Human Services, Research Triangle Park, North Carolina, USA

**Keywords:** communication, environmental exposure, occupational exposure, reproduction, research design, risk factors

## Abstract

The initial goal of occupational reproductive health research is to effectively study the many toxicants, physical agents, and biomechanical and psychosocial stressors that may constitute reproductive hazards in the workplace. Although the main objective of occupational reproductive researchers and clinicians is to prevent recognized adverse reproductive outcomes, research has expanded to include a broader spectrum of chronic health outcomes potentially affected by reproductive toxicants. To aid in achieving these goals, the National Institute for Occupational Safety and Health, along with its university, federal, industry, and labor colleagues, formed the National Occupational Research Agenda (NORA) in 1996. NORA resulted in 21 research teams, including the Reproductive Health Research Team (RHRT). In this report, we describe progress made in the last decade by the RHRT and by others in this field, including prioritizing reproductive toxicants for further study; facilitating collaboration among epidemiologists, biologists, and toxicologists; promoting quality exposure assessment in field studies and surveillance; and encouraging the design and conduct of priority occupational reproductive studies. We also describe new tools for screening reproductive toxicants and for analyzing mode of action. We recommend considering outcomes such as menopause and latent adverse effects for further study, as well as including exposures such as shift work and nanomaterials. We describe a broad domain of scholarship activities where a cohesive system of organized and aligned work activities integrates 10 years of team efforts and provides guidance for future research.

Data from the past decade underscore the public health relevance of studying workplace reproductive hazards. In 2003, Census data indicated that nearly 55% of children were born to working mothers (U.S. Census Bureau 2003a), and 62% of working men and women were of reproductive age (U.S. Census Bureau 2003b). The goal of occupational reproductive research is to effectively study the many toxicants, physical agents, and biomechanical and psychosocial stressors that may constitute reproductive hazards in the workplace. The difficulty of this task is compounded by several realities: the intrinsic methodologic limitations of both animal and human observational studies, the impact of mixed and multiple exposures, and complex work environments, both traditional and transitional. Although the main objective of occupational reproductive researchers and clinicians is to prevent recognized adverse reproductive outcomes, research has expanded to include a broader spectrum of health outcomes, such as breast cancer and the nature and timing of menopause and latent adverse reproductive effects.

The National Occupational Research Agenda (NORA) was formed in 1996, when the National Institute for Occupational Safety and Health (NIOSH) and its university, federal, industry, and labor partners unveiled the agenda as a framework to guide occupational safety and health research into the next decade. Approximately 500 organizations and individuals outside NIOSH provided input into the development of the agenda. The NORA process resulted in a remarkable consensus about the top 21 research priorities, including reproductive health research. The Reproductive Health Research Team (RHRT) included individuals engaged in basic laboratory research, epidemiology, risk communication, and public health and collaborated with other NORA teams engaged in exposure assessment, control technologies, and intervention effectiveness. In this report we describe a broad domain of activities that are relevant to public health applications.

## A Decade of Progress

The team’s initial achievements focused on prioritizing reproductive toxicants for further research, promoting the study of high-priority toxicants, and promoting occupational exposure assessment in existing surveillance studies. Recently, the team established a national occupational reproductive health research agenda (Lawson et al. 2003) to recommend future research directions to reduce the incidence of adverse reproductive health outcomes. This work can be accomplished with an interdisciplinary research program that identifies reproductive hazards, their mechanism of toxicant action, and target populations. To lay the groundwork for a better understanding of occupational reproductive health issues, the team sponsored a symposium on the clinical, epidemiologic, and exposure assessment aspects of occupational reproductive exposures at the 2003 Teratology Society Annual meeting (Grajewski et al. 2005).

### Prioritizing reproductive toxicants for further study.

Although more than 84,000 chemical compounds are in the workplace (2,000 new chemicals each year) (Endocrine Disruptor Screening and Testing Advisory Committee 1998), only about 4,000 have been evaluated for reproductive toxicity [U.S. Environmental Protection Agency (EPA) 1998]. Several NORA team members participated in the expert panel that prioritized chemical reproductive toxicants identified by the National Toxicology Program (NTP), using an objective and systematic method (Moorman et al. 2000). The method linked toxicity data with data on the population potentially at risk based on the estimated number of workers exposed and production data. Using this method, a priority matrix was developed that combined rankings for toxicity and number of workers at risk into categories of low, medium, and high. The panel found that the chemicals with the highest priority for human reproductive health studies were dibutyl phthalate, boric acid, tricresyl phosphate, and *N*,*N*-dimethylformamide. Chemicals with high/medium rankings included acrylamide, *N*-hydroxymethylacrylamide, 4-chloronitrobenzene, 2-butoxyethanol, oxalic acid, bisphenol A, and ethylene glycol.

Systematic prioritization of chemicals for study helps ensure efficient use of research funds. Many more chemicals remain to be studied, and the rankings should be periodically updated to incorporate new toxicity and usage data. Future priorities are likely to be affected by improved exposure information coming from biomonitoring data from the Centers for Disease Control and Prevention’s (CDC) National Health and Nutrition Examination Survey (NHANES).

### Promoting the study of prioritized toxicants.

Prioritized toxicants have been the focus of new studies initiated both inside and outside of NIOSH. Federal spending for occupational reproductive health research, in general, increased substantially and collaboratively during 1996–2003. For example, total NIOSH expenditures in the area of reproductive health increased from $750,000 in 1996 to > $4 million in 2004, for internal NIOSH research and for funding of research grants outside of NIOSH. As another example, an endocrine disruptor grants announcement was cosponsored by NIOSH, the National Institute of Environmental Health Sciences (NIEHS), the U.S. EPA, and the National Cancer Institute (NCI).

To promote the study of high-priority reproductive toxicants, the NORA team established a partnership with the Center for the Evaluation of Risks to Human Reproduction (CERHR). NTP and NIEHS established CERHR in 1998 to serve as an environmental health resource to the public and to regulatory and health agencies. Located in Research Triangle Park, North Carolina, the center’s staff and expert panel members represent multiple disciplines, including genetics, biology, toxicology, chemistry, industrial hygiene, biostatistics, epidemiology, and various medical specialties. The center provides scientifically based, uniform assessments of the potential for adverse effects on reproduction and development caused by agents to which humans may be exposed. This is accomplished through rigorous evaluations of the scientific literature by independent panels of scientists and through summarized reports in terms that can be understood by those who are not scientifically trained. Such evaluations encompass health effects including impaired fertility, adverse pregnancy outcomes, birth defects, and post-natal functional deficits. Nominations of chemicals to the CERHR are solicited from the public and the scientific community. Recent monographs on 1-bromopropane, 2-bromopropane, ethylene glycol, propylene glycol, and phthalates are available on the CERHR website (CERHR 2005b).

Internal NIOSH research is using field studies, exposure assessment, and laboratory biomonitoring to study several prioritized reproductive toxicants. One study is evaluating worker exposure to phthalate compounds, which are used as plasticizers and solvents in many industrial and consumer goods, such as flexible polyvinyl chloride, nail polish, fragrances, adhesives, and lacquers. In an NHANES report, phthalate levels were found to be elevated in the urine of women of reproductive age compared with levels for men (Silva et al. 2004). Several phthalates have demonstrated adverse reproductive effects, including male reproductive toxicity, in animals (CERHR 2000). There are virtually no published data available on the extent of phthalate exposures among working populations who use or are exposed to these chemicals, although thousands of workers may be exposed. Combining the field research expertise of NIOSH and laboratory expertise of the National Center for Environmental Health at the CDC, this project will measure levels of urinary metabolites of phthalates among workers in a variety of industries to identify populations for epidemiologic research.

Another NIOSH internal study is evaluating the extent of exposures to 1-bromopropane, a solvent and degreaser that is proposed to replace ozone-depleting solvents in metal and electronics industries. Potential dermal and inhalation exposure to 1-bromopropane can occur during metal degreasing, precision cleaning, and use of bromopropane-containing adhesives. 1-Bromopropane was nominated by NIOSH and selected for evaluation by CERHR based primarily on documented evidence of worker exposures and published evidence of male and female reproductive and developmental toxicity in rodents (CERHR 2004), although human reproductive studies were lacking. The exposure assessment consists of walk-through surveys, company record audits, industrial hygiene assessments (personal sampling as well as area monitoring), and measurements of exhaled breath (parent compound) and urinary metabolites. Another study is examining occupational exposure to acrylamide, used in the production of polymers and gels found in a wide variety of consumer products and as a cement binder. The NTP and CERHR have concluded that there is some concern for adverse reproductive and developmental effects from occupational exposure levels of acrylamide (CERHR 2005a). Workplace exposure monitoring, reproductive and neurologic health assessments, and biomonitoring will be conducted in the NIOSH study.

Boron is ubiquitous in nature and is used in a wide array of consumer goods. However, animal reproductive toxicity data and limited epidemiologic data indicate that boric acid and borax can cause reproductive toxicity in humans, and effects on sperm development have been observed in male animals (Moore et al. 1997). With funding from a NIOSH grant under NORA, investigators at the University of California at Los Angeles are collaborating with scientists in China to conduct a study of approximately 1,400 boron-exposed workers and unexposed workers in China. Laboratory measurements for this study integrated new sperm DNA integrity measures, conventional semen quality measures, hormones, blood-urine-semen boron, and boron levels in food, drinking water, and the workplace. Currently, analysis of data and biologic specimens is continuing.

Although 3% of babies in the United States are born with a major birth defect (CDC 1995), the cause of > 40% of birth defects remains unknown (Holmes 1997). Traditionally, few etiologic studies of birth defects have addressed parental occupational exposures, even though thousands of chemicals are being used in the workplace by men and women of reproductive age. To have a sufficient sample size to conduct research on specific types of birth defects, it is important to have collaboration among multiple research sites. The CDC has established the Centers for Birth Defects Research and Prevention in Arkansas, California, Iowa, Massachusetts, North Carolina, New Jersey, New York, Texas, and Utah. These centers, along with the Metropolitan Atlanta Congenital Defects Program, have participated in the National Birth Defects Prevention Study (NBDPS), the largest case–control study of birth defects ever undertaken. NIOSH scientists are collaborating with the CDC National Center on Birth Defects and Developmental Disabilities and NCI to conduct an occupational exposure assessment using parental occupational information collected as part of the NBDPS. Parental exposures to solvents, metals, and pesticides will be analyzed, and estimated exposure among cases and controls will be compared.

## Looking Forward: The Next Decade of Occupational Reproductive Research

### Rethinking outcomes and exposures.

The changing nature of work and the work environment and the emerging technologies in reproductive biology and exposure assessment are leading us to rethink approaches to studying exposures and traditional reproductive health outcomes. It remains important to emphasize that the spectrum of reproductive health outcomes includes not only women of childbearing potential but also all working women, all working men, and all of their potential offspring. Clinical outcomes among workers should include sexual dysfunction, infertility, pregnancy loss, male:female sex ratios of pregnancies, aberrations of fetal growth, preterm births, clinical manifestations of endocrine disruptions (e.g., early menopause and andropause), and reproductive organ and endocrine-mediated neoplasms. Outcomes among offspring include congenital malformations, developmental challenges, infant and childhood neoplasms, and potentially adult reproductive health outcomes.

An example of an emerging end point for the assessment of adverse reproductive health in women is entry into the menopausal transition. In addition to providing a marker of ovarian senescence, the transition to menopause marks the beginning of a series of hormonal changes of biologic and clinical importance. Both early and late menopause are well established as associated with chronic health challenges (Gordon et al. 1978; Lindquist et al. 1979; Trichopoulos et al. 1972). A more recent study suggests that menopause is associated with a decline in grip and pinch strength (Kurina et al. 2004). From a research perspective, a standard definition of the start of the menopausal transition would allow important comparisons across occupational health studies; some efforts have been made in this area (Lisabeth et al. 2004). Age at menarche, although a more clearly defined end point, has been found to be associated with environmental exposures, including lead (Selevan et al. 2003).

Nonstandard work hours may be disturbed by many physiologic functions and systems that are circadian in nature (Akerstedt 1990). Circadian rhythms normally occur in the reproductive endocrine system (Frazier and Grainger 2003). Thus, hormonal disturbances, either as a direct effect of circadian rhythm disruption or indirectly through psychosocial stress and altered sleep patterns, are suggested as a possible mechanism. The effect of shift work, and circadian rhythm disruption, on reproductive outcomes is poorly understood, although advances have been made in the development of metrics for measuring disruption of circadian rhythm in working populations. One such metric is the variability of 2-sulfoxymelatonin, the urinary metabolite of melatonin, which has been found to be correlated with travel by female flight attendants through multiple time zones (Grajewski et al. 2003).

To better understand the impact of shift work and long work hours on reproductive health, important data may be leveraged from ongoing prospective studies. In 2001, with NORA funding, NIOSH investigators initiated a collaborative study with the Harvard University Nurses’ Health Study II (NHS II) research team. This study is collecting and analyzing data from 10,000 members of the NHS II cohort. Before this study, few occupational data had been collected from the NHS II cohort. A successful, ongoing collaboration was developed between NIOSH and Harvard that will likely engender consideration of the effects of other occupational exposures on reproductive health.

One of the most promising prospective studies that will add to our understanding of reproductive health is the National Children’s Study (NCS), a multiagency landmark study of 100,000 children from preconception to adulthood (National Children’s Study 2005). NIOSH NORA team members have partnered with NCS planners to provide guidance on how to include parental occupational histories as part of the baseline metrics of the cohort. This project will allow many hypotheses to be tested regarding parental exposures and their impact on congenital anomalies, developmental delays, sexual differentiation, puberty, and subsequent fertility.

An area that merits further exploration through NCS and other studies is the relationship between parental occupational exposures, fetal growth, and distant postnatal health. For example, according to the Barker hypothesis (Khan et al. 2003; Lau and Rogers 2004), low birth weight increases the risk of obesity, hypertension, and cardiovascular disease during adulthood. Because exposure to developmental toxicants is associated with low birth weight, research is being directed at testing the Barker hypothesis with regard to toxicant-induced low birth weight. Preliminary evidence indicates that low birth weight per se (i.e., that due to controlled underfeeding during pregnancy) is not associated with adverse reproductive capacity in the offspring (Rogers et al. 2003). Continuing research will determine whether toxicant-induced low birth weight has the potential to affect reproductive capacity and other health outcomes of offspring.

Rethinking occupational exposures merits consideration of the emerging field of engineered nanomaterials [uniformly sized materials < 100 nm (1 nanometer = 10^−9^ meter)]. Nanotechnology is being touted as a great opportunity for technologic advancement, but the potential toxicologic hazards associated with the increasing commercial applications of nanotechnology are still being characterized (Colvin 2003). The National Nanotechnology Initiative, officially established in fiscal year 2001 (National Research Council 2002), involves 17 federal agencies, including NIOSH. The private sector is also actively involved in nanotechnology research, and applications for computer components, cosmetic products, textiles, medical imaging, and drug delivery are already in commerce or under development (Perkel 2003, 2004). Nanoscale zinc oxide and titanium dioxide, for example, are both currently being incorporated into sunscreen lotions (Royal Society and Royal Academy of Engineering 2004), and as a result, both production workers and consumers (including pregnant women) are potentially exposed to these materials. Colvin (2004) suggested that by changing the surface properties, engineered nanoparticles can cross cell membranes and potentially circulate in the blood. Hence, it is theoretically possible that nanoparticles may cross the blood–brain barrier and the placenta. Because exposures to men and women and children may already be occurring, there is a clear need to investigate the potential reproductive health risk of engineered nanomaterials (Dreher 2004).

Another challenge in consideration of occupational reproductive health is assessment of multiple exposures. If workers are exposed to multiple compounds that act by the same mechanism, effects may be additive or synergistic, even though no single exposure occurs above occupational exposure limits (NIOSH 2005). This concern is supported by toxicologic studies showing additivity of adverse reproductive effects from solvent mixtures (Brown-Woodman et al. 1994), anti-androgenic fungicides (Nellemann et al. 2003), metals and chlorinated hydrocarbons (Roegge et al. 2004), and other mixtures. For this reason, it has been standard industrial hygiene practice to use a mixture formula to calculate a lower acceptable occupational exposure level when multiple exposures occur in the work-place [American Conference of Governmental Industrial Hygienists (ACGIH) Worldwide 2004]. More research is needed on mechanisms of toxicity to determine when this risk assessment procedure should be applied. Even more challenging may be consideration of the effects of physical hazards in concert with chemical hazards; for example, whole-body vibration can affect androgen levels just as chemical toxicants can (Cardinale and Pope 2003). Interpreting available information on additive and synergistic effects of exposures remains a challenge for employers, especially small businesses with limited access to industrial hygiene and toxicologic specialists. It is incumbent on occupational health researchers and policy makers to address these challenges to better protect all workers.

### Basic research and new tools.

#### Opportunities for high throughput and customized screening.

One of the most significant issues in the regulation of toxic compounds has been the gap between the number of chemicals that are in commerce and the number that have been thoroughly tested for their ability to affect reproduction and development. The reason for this gap is that the current state-of-the-art in toxicity testing consists of protocols in laboratory animals that are time and labor intensive. Even though the test species have been selected, in part, because of their short reproductive cycles, these cycles take several months to complete, so a full assessment of a chemical may take a year or more. There are a number of possible ways to screen chemicals for prioritizing for future testing, including quantitative structure–activity relationship predictions, high-throughput screening for a specific biologic activity, or *in vitro* assays that mimic one or more critical biologic events that occur as part of the reproductive process.

One of the impediments to using these screening techniques is that their development and effectiveness depend on having a good understanding of the key biochemical and molecular events that control reproduction and development and that may be the targets of toxicants. Continuing advances in our understanding of this underlying molecular control are making it possible to design structure–activity relationship programs and high-throughput screens that may be useful for prioritizing compounds based on putative mechanism of action and potency. High-throughput screening assays are already being used in the pharmaceutical industry, where the process of drug development involves screening tens of thousands of compounds at a time for therapeutic efficacy and possible toxicity. It is clear from these efforts that high-throughput screening for identifying potential toxicants is feasible (Meador et al. 2002; Waring and Ulrich 2000).

The sex steroids (androgens, estrogens, and progestogens) have long been known to be important to reproduction, but recent advances in science have made it possible to create practical screening assays that have a remarkable degree of specificity. High-throughput assays for estrogen receptor binding are commercially available, using recombinant forms of the human estrogen receptors. There are a large number of reporter gene assays for estrogen and androgen receptor activity [Interagency Coordinating Committee on the Validation of Alternative Methods (ICCVAM) 2003]. Structure–activity relationship programs have been developed for estrogens (Blair et al. 2000; Bradbury et al. 2000; Mekenyan et al. 2000) and androgens (Fang et al. 2003; Singh et al. 2000). These assays hold the potential to evaluate the binding affinity of large numbers of compounds, which would then be subjected to more extensive screening. In addition to these receptor binding assays, research is proceeding to develop cell-based assays to identify inhibitors of steroidogenesis, a non-receptor-mediated mechanism of endocrine toxicity (Hilscherova et al. 2004). Initiatives are underway to systematically develop *in vitro* assays for all aspects of the reproductive cycle.

Because the output of the screening-level assays can only be evaluated in a limited number of animal study designs (all time- and resource-intensive), there is a need to rethink the way that we approach chemical testing. Specifically, the assessment program for any given chemical could be customized such that the testing is focused on the most likely outcomes of the potential mechanism(s) of toxicity, identified in the screening level. This may lead to tiering of testing, such as the U.S. EPA’s Voluntary Children’s Chemical Evaluation Program, which uses an iterative analysis of toxicity and exposure information to determine when more data are needed to adequately characterize a chemical’s risk to children (U.S. EPA 2005).

Another area of considerable research activity is the development of quantitative structure–activity relationship (QSAR) software to predict the potential of a chemical to have a specific biologic activity, either through comparison of its chemical structure with that of a series of related chemicals or by using physical chemical parameters to determine the likelihood and affinity of the chemical binding to a particular receptor. QSAR models will continue to be developed for screening large numbers of compounds for their ability to interact with specific biologic receptors.

#### Understanding mode of action.

Much of the basic science of toxicology is being devoted to understanding the modes of action by which exogenous agents affect living systems. Mode of action information is important for a variety of reasons, including *a*) supporting the validity of predicting human risk from data generated in animal models; *b*) serving as the basis for extrapolating data from those models for making quantitative predictions of human risk; *c*) elucidating common mechanisms of action among different toxicants, thereby supporting additive risk assessment for mixtures; and *d*) supporting the biologic plausibility of associations between exposures and adverse effects.

Although mechanistic research is not new, the pace of progress is likely to accelerate significantly with the advent of genomics (especially functional genomics or “transcriptomics”) and the related fields of proteomics and metabonomics. Functional genomics involves a genomewide evaluation of the changes in gene expression in response to a perturbation. In some instances the transduction of the exogenous signal involves gene expression; in other cases the gene expression represents the cell’s attempt to regain homeostasis. Functional genomics provides a comprehensive look at these responses and important clues as to modes of action, clues that are used to formulate hypotheses for further testing.

In reproductive toxicology, much of the work using genomics technologies has been in the area of endocrine disruptors. Naciff et al. (2002) have identified the genes in the fetal rat uterus and ovaries that are responsive to estrogens, as a means of cataloging the possible candidate genes whose altered expression may lead to the latent, persistent effects that have been observed after developmental exposure to potent estrogens (Naciff et al. 2004). Others have evaluated the time course of gene expression during the estrous cycle in mice (Fertuck et al. 2003), as a means of determining the genomic control of this physiologic process. In the course of this work, a number of genes that were not previously known to be estrogen responsive have been identified. Importantly, it has been determined that, generally, all binders to the estrogen receptor act in the same manner at a molecular level (Moggs et al. 2004; Naciff et al. 2002, 2003), although the number of compounds tested thus far is small compared with the number of compounds with potential to bind to the several subtypes of estrogen receptors.

Other research has evaluated the effects of various toxicants on patterns of gene expression in embryos, in an attempt to elucidate mechanisms of abnormal development. Hunter and colleagues (Simmons et al. 2002) have identified a series of genes that are responsive to chlorinated by-products of drinking water disinfection and that may be related to the congenital cardiovascular defects associated with high-dose exposures to some of these compounds. Knudsen’s laboratory (O’Hara et al. 2002) has used genomics to identify mitochondrial metabolism as the potential target for mercury. Tully et al. (2004) report changes in testicular gene expression profiles in rats exposed to bromoacetic acid, a disinfection byproduct in drinking water.

In sum, this research area is likely to elucidate a number of questions relevant to characterizing workplace reproductive hazards. Because it appears that the pattern of gene expression is characteristic of a particular mechanism of action, this technology may be useful in identifying agents with a common mode of action for the purpose of conducting cumulative risk assessment. Also, because gene expression changes precede frank toxicity, it may be possible to use gene expression data as a means of predicting latent health effects.

#### Use of new technology for gene/environment interactions.

With the publication of the initial draft of the human genome (International Human Genome Sequence Consortium 2000; Venter et al. 2001), there has been greater awareness of the role of genetics in affecting individual responses to environmental chemicals. The number of variants, mainly in the form of single nucleotide polymorphisms (SNPs), within the human genome is estimated to be > 1.4 million (International SNP Map Working Group 2001), although not all of these SNPs are localized within functional genes. An example of gene–environment interactions influencing reproductive outcome is the association between polymorphisms in the paraoxonase gene, an enzyme that has been linked to risk of preterm delivery and other end points (Chen et al. 2004). Other examples of polymorphisms associated with adverse effects on reproductive functions include the association of *CYP1A1 Msp1* polymorphism, which codes for a P450 enzyme involved in the detoxification of various environmental toxicants, with increased risk of low birth weight (Chen et al. 2005), and the association of *HER2 I655V* polymorphism, which codes for a transmembrane glycoprotein with tyrosine kinase activity that is involved in regulating cellular proliferation, with increased breast cancer risk in women < 40 years of age (Montgomery et al. 2003).

The Environmental Genome Project (EGP) was initiated within the NIEHS in 1997 to examine how genetic differences among individuals affect disease risk from environmental agents. The EGP is concentrating on approximately 200 environmentally responsive genes, with primary focus on the SNP or single-base variation that occurs at a frequency of at least 1% of the population (Brookes 1999). There may be multiple SNPs for each typical gene (Cargill et al. 1999); therefore, it will be important to characterize the specific functional change resulting from the SNP so that an association can be made between exposure to the chemical of interest, sequence variation, and altered response. The EGP will be challenged to select which SNPs to evaluate in field studies, which chemical exposures to assess, and how to quantify these exposures. Because exposure measures for most of the chemicals of interest are limited, there is a critical need to quantify internal dose levels for potential human reproductive toxicants.

Although many studies have reported the association of various polymorphisms with altered response to environmental toxins, these studies are limited by the modest level of association between the polymorphism and exposure (Ioannidis et al. 2001). There is also a lack of reproducibility in many gene–environment association studies (Hirschorn and Daly 2005) that may be due to multiple risk factors associated with the outcome and multiple genes controlling the susceptibility (Blangero 2004). Thus, future studies will require the careful selection of the study population and of the candidate polymorphism(s), accurate estimates of exposure to a toxin(s) to identify associations (Tabor et al. 2002), and adequate study populations to provide sufficient statistical power (Hunter 2005). In addition, genomic studies have ethical and social implications that need to be considered, such as insurance and employment discrimination, stigmatization, and privacy issues in the occupational setting (Burke et al. 2002).

#### Computational analyses.

A major challenge for making use of new technologies is to integrate and interpret genomic/proteomic/metabanomic information with toxicologic and epidemiologic end points. Currently, standard reproductive health end points (e.g., sperm motility, hormone measures) can be used as markers of reproductive health outcomes in humans; however, the extent to which changes in gene expression (proteins/ metabolites) predict adverse reproductive effects remains to be characterized. The success of this effort will rest on development of well-designed toxicogenomic databases. International and interagency efforts are underway to this end (Mattes et al. 2004).

### Communication.

Developing and providing effective communication is a major challenge within the public health and occupational health communities. Workers in multiple industries need clear, quickly accessible information that can advise men and women on risks to their reproductive health. The NIOSH NORA RHRT has collaborated with the Hazardous Drug Working Group to update written instructions and label warnings for certain hazardous drugs. The RHRT is also interested in finding ways to improve the quality of material safety data sheets (MSDSs), with special interest in improving the quality of reproductive health information.

Reproductive risk communication research is needed for the development of effective ways to communicate with workers about occupational reproductive hazards. For men, the extent of risk minimization (the belief that men are not susceptible to reproductive hazards) needs to be determined. Among women, methods are needed to improve communication about the importance of exposure reduction in the preconception and periconception periods.

Effective hazard communication programs translate technically complex terms from reproductive health research into language that workers can easily understand (“plain language”). Despite extensive literature on the comprehensibility of educational materials for topics such as nutrition, smoking cessation, and cancer treatment, there is little published research on the comprehensibility of materials used for workplace hazard communication. In one study, 100 workers from manufacturing industries were asked to read several MSDSs written at the 12th grade reading level (Kolp 1993), and then their understanding of this information was tested. Of a possible score of 100 points, comprehension scores were in the range of 60–67, suggesting that a third of the MSDS health and safety information was incomprehensible to workers. One-fourth of the adult population in the United States has limited literacy skills (American Medical Association 1999), and it is recommended that health education materials should be written at the 5th to 8th grade reading level (National Work Group on Literacy and Health 1998). The American National Standards Institute (ANSI) devotes several pages of its revised standard on MSDS preparation to communication principles, providing good general guidelines on techniques to enhance comprehensibility of these important documents (ANSI 2004). The NORA RHRT conducted a session on MSDS communication at the 2005 Society of Toxicology Meeting to help improve reproductive hazard communication.

Also needed is consensus building on how best to classify reproductive hazard data for occupational health communication. This could assist occupational health and safety professionals to use best practices when writing MSDSs or designing occupational hazard communication programs. To account for different levels of evidence, the Globally Harmonized System of Classification and Labeling of Chemicals provides three classification categories for reproductive toxicants—known, presumed, or suspected reproductive or developmental hazards—and also a category to designate effects on, or via, lactation (Silk 2003; UN Economic Commission for Europe 2003). Detailed methods for interpreting toxicologic and epidemiologic research are provided, and concentration limits trigger classification of a mixture into each hazard category. For each of these categories, a hazard statement written in nontechnical language is provided. This initiative emphasizes the importance of testing communication materials for comprehensibility (Silk 2003). Development of risk-based classification information is preferable to a category approach but will take consensus efforts to implement. Future research needs to apply methods such as these to ascertain the perceptions of workers with varying levels of literacy and differences in cultural experiences and to determine their effectiveness in promoting safe work practices.

## Research to Practice in Occupational Reproductive Health Research: The Case of Hazardous Drugs

NIOSH’s new Research to Practice (r2p) initiative is designed to transfer research findings, technologies, and information into effective prevention practices and products and to promote their adoption in workplaces. The goal of r2p is to decrease occupational illnesses, injuries, and fatalities by increasing the work-place use of effective NIOSH and NIOSH-funded research findings.

A vibrant example of r2p implementation is the NORA RHRT’s Hazardous Drug Working Group activities regarding the unsafe handling of hazardous drugs in health care settings, an instance in which exposure opportunity is unregulated and the hazard is high. Scientific evidence appeared in the literature several years ago documenting widespread contamination of oncology clinics and pharmacies with antineoplastic hazardous drugs in a number of university hospitals in the United States and Canada (Connor et al. 1999). Although the Occupational Safety and Health Administration (OSHA) and professional organizations of hospital pharmacists and oncology nurses have published safe handling guidelines (OSHA 1986, 1995), it was apparent that, despite the high potential health risk these drugs posed to workers when handled improperly, there was poor adherence to recommended standards of safe professional practice (Connor et al. 1999). The existing gaps in the collective science include full toxicologic characterization of these drugs in health care exposure settings, industrial hygiene methods to describe exposure, adequate risk communication to affected workers, and vigilance in assuring and evaluating safe handling work practices. This single example is summarized according to the Carnegie scholarship model of discovery, integration, application, and teaching (Boyer 1990) in Table 1.

Discovery scholarship—that is, new knowledge—continues to accrue and, in so doing, suggests further information gaps regarding these most toxic therapeutic drugs, many of which are known carcinogens and undisputed human reproductive and developmental toxicants. Engineering scientists are collaborating with toxicologists on the hazardous drug problem in a relevant example of integration scholarship, the making of connections across disciplines. For example, the fugitive drug particulate captured on high-efficiency particulate air (HEPA) filters in biologic safety cabinets appears to be volatilizing under the high-volume air flows passed over the filters. This volatilization presents a potential exposure to workers not currently addressed elsewhere. There is a cascade of implications for change in both engineering controls and work practices if this volatilization exposure is found to be commonplace.

The scholarship of application describes a lively engagement between affected parties in the sciences and the beneficiaries of that science to fully use the knowledge gained from both the discovery and integration activities. As an example, NORA RHRT partnered with the NORA Control Technology Team to sponsor and support a working group to review the new evidence regarding ongoing exposure of health care workers to hazardous drugs and to assess the need for change of current work recommendations. The Hazardous Drug Working Group is composed of stakeholders from all federal government agencies and regulatory bodies affected, health care worker unions, professions, home care providers, drug manufacturers, and academia. The group reviewed new evidence of exposure against existing OSHA and professional practice guidelines to determine where gaps exist in compliance and the worker knowledge base. This group produced a NIOSH Alert (NIOSH 2004) titled “Safe Handling of Hazardous Drugs in Healthcare,” which served as an organizing focus for stakeholders to brainstorm and translate this new science base into specific work practice applications, identifying strategies to improve safe handling and enhance worker protection.

Teaching scholarship, extending and communicating knowledge to the affected public, began in this r2p effort with a health care industry “rollout” workshop in October 2004 (Alert on Reducing Occupational Exposures to Hazardous Drugs in Healthcare: Converting Theory to Practice, 3–5 October 2004 , San Antonio, TX) to raise national awareness about hazardous drug exposures and provide the scientific base for the Alert. NIOSH, OSHA, and the Joint Commission on Accreditation of Healthcare Organizations leadership opened the meeting to call the attendees to action on the part of the workers they employ. Work group members presented sessions offering real-world practical solutions to support attendees in applying new information to improve their organization’s safe handling programs.

## Summary

Effectively studying the many toxicants, physical agents, and biomechanical and psychosocial stressors that may constitute reproductive hazards in the workplace is challenging. NIOSH’s NORA RHRT has implemented several approaches to improve occupational reproductive research: prioritize reproductive toxicants for further study; promote analysis of occupational exposure assessment in reproductive health surveillance; facilitate collaboration among epidemiologists, biologists, and toxicologists; promote quality exposure assessment in field studies; and encourage the design and conduct of priority occupational reproductive studies. Here we describe new tools for screening of reproductive toxicants and for analyzing mode of action. We recommend considering outcomes for further study such as menopause and latent adverse effects, as well as including exposures such as shift work and nanomaterials. This report describes a broad domain of scholarship activities where a cohesive system of organized and aligned work activities integrates 10 years of team efforts and provides guidance for future research.

## Figures and Tables

**Table 1:** Domains of scholarship of the NORA RHRT: hazardous drugs as an example.

Domain of scholarship^a^	Description	Example from the Hazardous Drugs Working Group^b^
Discovery	Traditional research NIOSH laboratory and field studies	Studies of hazardous drugs as reproductive toxicants: industrial hygiene, biologic monitoring, and protective apparel research
Integration	Making connections across disciplines Interdisciplinary research teams	Engineering and toxicology collaboration to study volatilization of hazardous drug particulates from laboratory hood filters
Application	Applying research knowledge to consequential problems	Reproductive health and control technology NORA teams collaborate to apply research knowledge to hazardous drugs, resulting in the formation of the working group and publication of the NIOSH Hazardous Drugs Alert
Teaching	Communicating Transforming and extending knowledge	Hazardous Drugs Alert “rollout,” including training and outreach Annual update of information

a As described by Boyer (1990).

b A NORA working group that examined handling practices for hazardous drugs and promoted safe handling of these substances. To address these needs, a NIOSH Alert on Occupational Exposure to Hazardous Drugs was developed (NIOSH 2004), and a national workshop was held in 2004.
